# The Role of CoLab and GAITS in Enabling the RADx Tech Program

**DOI:** 10.1109/OJEMB.2021.3070829

**Published:** 2021-04-28

**Authors:** John M. Collins, Marshall R. Collins, Tamara McKenzie, Mark Marino

**Affiliations:** 1 Center for the Integration of Medicine and Innovative Technology (CIMIT)Massachusetts General Hospital and Harvard Medical School Boston MA 02114 USA; 2 BiocomX Dana Point CA 92629 USA; 3 VentureWell 263310 Hadley MA 02114 USA

**Keywords:** CoLab, commercialization, GAITS, healthcare innovation, RADx Tech

## Abstract

The RADx^SM^ Tech initiative required a massive mobilization of the biomedical community. It was chartered with the extremely ambitious goal of rapidly developing and deploying innovative tests to detect people infected with the SARS-CoV-2 virus. It needed to do so at a scale and with urgency to get the country back to daily activities such as school and work as soon as possible. It required forming and supporting a diversity of teams with members from around the country and beyond. These teams collaborated in complex workflows that needed to be carefully monitored and tracked. This paper describes the key elements of the secure, web-based infrastructure that was configured to enable the efficient and effective operation of RADx Tech's key processes and address its unique and urgent challenges. One such challenge was to manage the flow of applications through a multi-stage, interactive selection process (using the CoLab platform) and another was to support and facilitate the progress of projects selected for support and funding through an accelerated commercialization program (using the GAITS platform).

## I. Background

As explained by Dr. Bruce Tromberg, (Director of the National Institute of Biomedical Imaging and Bioengineering – NIBIB) in his opening webinar to the RADx Tech Faculty (May 26, 2020), by mid-April 2020 SARS-Cov-2 testing was plateauing at about 150K tests per day. This was a real concern for many people since testing was recognized as a key source of objective data for the medical community to monitor the spread of COVID-19. Dr. Tromberg explained that he was invited to a call with Dr. Francis Collins (NIH Director) and Senator Lamar Alexander (R-Tenn. and chairman of the Senate Health, Education, Labor and Pensions Committee) to talk about the work already ongoing in the Point of Care Technology Research Network (POCTRN^1^) to repurpose tests.

Senator Alexander then wrote a “Shark Tank” opinion piece^2^ with Senator Roy Blunt (R-Mo. and chairman of the Senate's health appropriations subcommittee). In the piece, they proposed “a competitive “shark tank” — much like the reality–TV show about entrepreneurs, but this time utilizing the capacities of government itself, in coordination with the private sector, to pull out all the stops and create new technologies designed to produce tens of millions of diagnostic tests per day.

Soon after the Washington Post Op Ed was published, $1.5B was appropriated by Congress to NIH, out of which $500M was allocated to NIBIB to rapidly develop and deploy SARS-CoV-2 diagnostic tests in a program subsequently named RADx^SM^ Tech. On April 19th, 2020, POCTRN was chosen by NIBIB to lead RADx Tech, with CIMIT^3^ as the Coordinating Center. Since the launch was 10 days later, the RADx Tech initiative needed to be implemented immediately to respond to the aggressive targets and be able to deliver timely results.

## II. Virtual Infrastructure Needs and Challenges

While POCTRN was an operating program, it needed to reinvent itself to handle the challenges represented by RADx Tech. It needed to build on its virtual infrastructure to operate at scale as quickly as possible and morph itself to address the many unique needs and challenges faced at the outset of the RADx Tech program. Key among the challenges were managing workflows and facilitating the collaborative contributions of hundreds of people from different organizations that would need to work together, most of whom for the first time.

*Workflow Management:* The need was to create rigorous, secure, scalable, and traceable workflows that could:

1) Be implemented quickly and be adaptable as the situation evolved so that previous workflows could be adjusted, or new workflows could be created and implemented.
2) Be scalable since the magnitude of the response to the call for applications made public on April 29, 2020 could not be known in advance.
3) Facilitate collaboration securely across multiple institutions and individuals.
4) Be simple and require little to no training.
5) Provide real-time reporting to the RADx Tech and NIBIB leadership on projects and time spent by person.
6) Have traceability to RADx Tech funding recommendations/ justifications and related NIH decisions.
7) Capture all the data in a secure manner to enable analysis and ad-hoc reporting as well as help extract quantifiable lessons learned.

As explained below, this was accomplished by reconfiguring and significantly expanding CIMIT's CoLab platform (built on the Open Water Grants Management Software^4^), which CIMIT had already used to manage the POCTRN solicitation process among many other uses.

*Facilitating Project Collaboration:* As explained in more detail below, RADx Tech needed to run more than 120 “shark tank”-like sessions and simultaneously manage about 50 commercialization projects with experts in various fields who, most often, had not previously worked together before. As such, it was critical that RADx Tech find a way to establish a common language and development framework as well as a secure information infrastructure that provides:

1) A way for the hundreds of people recruited to work together in providing support to the applicant teams to assess the status of a project, develop work plans, and keep the status updated in a consistent and trackable way.
2) An information portal that enables access to project, portfolio, and program content to approved individuals on a secure, need-to-know basis.
3) RADx Tech leadership and the NIH a view of the entire portfolio and sub-portfolios of projects as well as active work packages with the ability to drill-down into any level of detail desired.
4) A way to capture project-specific content generated during the work (presentations, quad charts, etc.) as well as output metrics created by funded projects.

As explained below, this was accomplished with CIMIT's Guidance and Impact Tracking (GAITS) platform^5^, a secure, web-based platform based on CIMIT's 20+ years of experience in helping teams successfully navigate and accelerate the challenging journey of innovation in healthcare.

*Other Software Platforms:* In addition to the unique needs of RADx Tech, teams also needed other software platforms to effectively collaborate and conduct their work. RADx Tech core partner, VentureWell, was responsible for managing the availability of the following software platforms used by teams: Zoom Business, LucidChart Team, Microsoft Project Plan 3, Smartsheet Business, Greenlight Guru, and Slack. They also managed the time-card management process through the ClickTime platform, described in more detail below.

## III. CoLab and Workflow Management

RADx Tech required several distinct workflows to operate in parallel. The key ones implemented in the CoLab platform were:

• *Fast-Track workflow:* The main workflow is that for applicants to apply and then have that application move through the series of steps and reviews^5^. The unique elements of the Fast-Track Workflow implemented in the CoLab platform will be outlined in more detail below.
• *Expert enrollment:* An expert enrollment workflow was used as part of an outreach program to find the diversity of experts needed to support teams. Expertise and willingness to play one or more of the roles was captured along with a characterization of the expertise and available time as well as machine readable bio sketch. The data were used later find people with expertise needed to support a team as a member or to address a specific question. It also provided the raw data to be used in the timecard reporting platform described below. At the time of this writing, more than 560 experts had been recruited with more than 220 engaged through RADx Tech contractual centers (BiocomX, CIMIT, University Lab Partners, or VentureWell)
• *Specification development:* In evaluating potential diagnostic test solutions, it was recognized that different use cases required different specifications: there was no single specification that all technologies needed to achieve. For example, an at-home test can take longer to achieve a result than a test at an airport, which could also have a higher false-positive rate. A survey of the medical experts was conducted and analyzed to help inform the RADx Tech team in developing specifications that would be used for a small number of target use cases.
• *Conflict of Interest (CoI) management*: This surfaced during the program as the NIH required an executed CoI statement for each person working on an RADx Tech-related effort. CoLab was needed to manage the process and provide traceability since the agreements that individuals had with their own institution varied.
• *Vendor and service provider validation*: As the projects moved from the initial Deep Dive review with a team of experts to the active Work Packages (see article by Dempsey et al. and Gagliano et al. in this special issue), the level of support they needed required the resources available through companies rather than by individual experts. The need was for an approved list of vendors and suppliers that had been vetted by RADx Tech and approved by the NIH so that they could be quickly brought on to support teams as well as track the work done and satisfaction for future reference. This process was managed by VentureWell. At the time of this writing, more than 74 vendors and service providers were reviewed and approved, with more than 80 scopes of work developed.
• *Timecard and vendor management*: To add additional functionality to CoLab, VentureWell, one of the core organizations supporting RADx Tech, provided the virtual infrastructure (ClickTime^6^) to monitor, track, and approve the time spent by experts in supporting teams or in performing work in support of RADx Tech^7^. Data to populate the database came from the CoLab expert enrollment workflow.

**FIG. 1.**
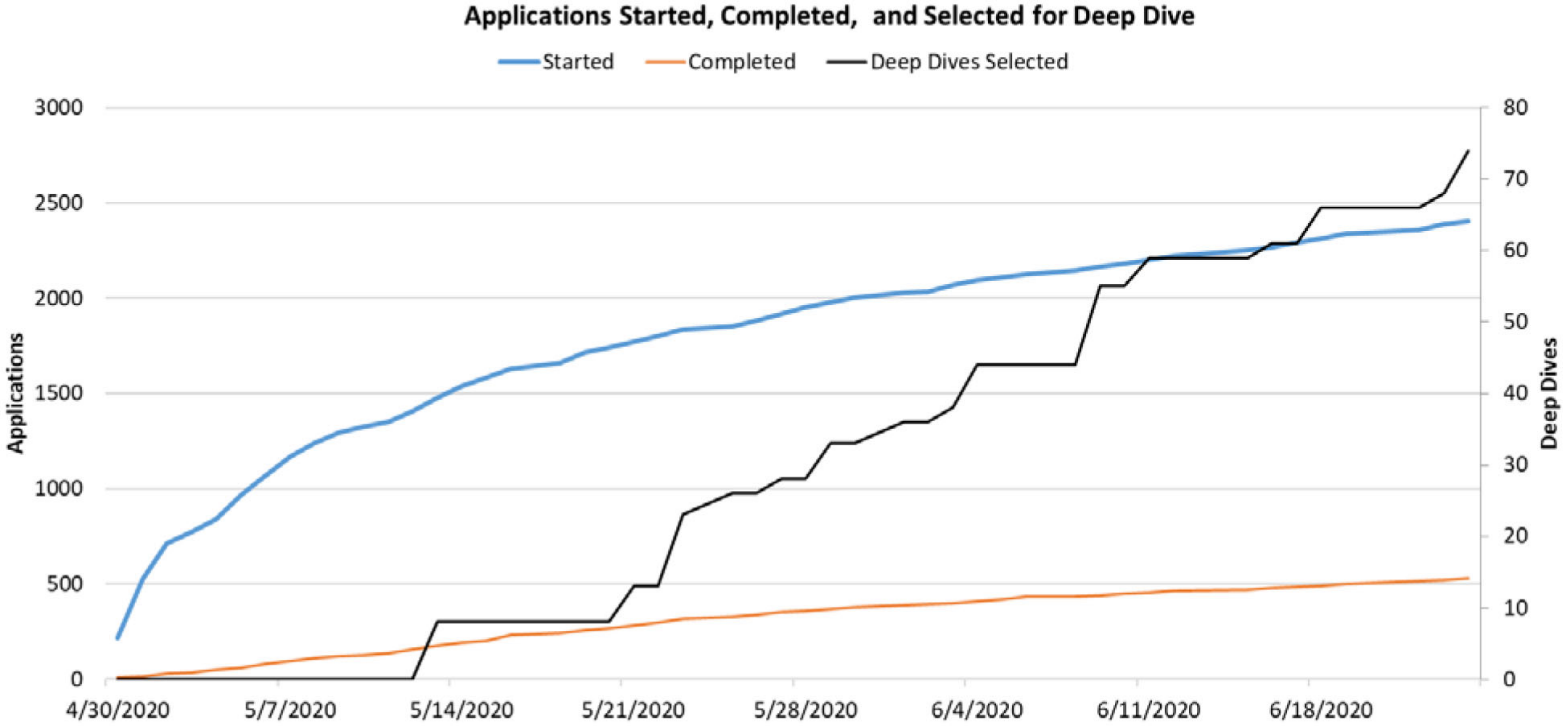
Cumulative totals through June 2020.
• *RADx Tech SWAT Team collaboration:* A core premise of RADx Tech was to support and augment project teams so they could focus their time as much as possible on advancing the technology and not writing proposals and reports. As such, the RADx Tech teams assembled to support each project (called SWAT Teams) were given the responsibility to input the data requested in each of the subsequent CoLab steps. This was a very important and unusual transition in the Fast-Track workflow. Steering Panel members and NIH program officers who reviewed the presentation materials knew that they were prepared by an objective team of experts. This enabled a consistent way for projects to be presented and allowed Steering Panel members to focus on the potential of the technology to meet the RADx Tech objectives rather than attempting to “read between the lines” of applications written and presented by project team members trying to put their application in the best possible light.
• *Transparency and access:* Given the high interest in the RADx Tech solicitation, resulting in large numbers of applications coming in daily, it was necessary to collect and act on final NIH decisions quickly and accurately. With multiple stages, and possible decision types, it was important to implement a robust workflow to not to lose track of decision status as can easily happen using typical methods such as a shared Google doc. With existing out-of-the-box functionality, the NIH set up a connection with Open Waters Representational State Transfer Application Programming Interface (REST API). This allowed faculty at the NIH to directly access the application review comments and recommendations as well as input decisions to CoLab without the need to learn a new system. It also allowed the CoLab manager to act on decision functions in real time. The API further permitted the NIH to pull in metadata to build their own dashboard and reports that are kept behind NIH firewalls. The REST API calls for NIH decisions corresponding with a separate stage of the workflow that each application entered at some point prior to receiving any notification.

*Fast-Track Workflow:* The Fast-Track workflow of innovative testing technologies was a core element of RADx Tech and required some novel approaches, which made it extremely effective. Some of the key attributes that were implemented included:

• *Real time monitoring:* RADx Tech operated in moving innovations through the Fast-Track process in a rolling manner in near real-time to minimize delays. It was therefore critical to monitor the rate at which applications were started and completed as well as how they moved through the subsequent steps of review and work while in funded stages (Deep Dive, WP #1, and WP #2) to be able to plan the staffing capacity needs. To provide a scale of response, Fig. 1 below shows the cumulative totals for applications started, completed, and selected for Deep Dive from the program start in late April through June 2020. At the time of this writing, more than 2800 applications were started and 700 completed.

## IV. GAITS and Project Facilitation

Experience with the POCTRN program demonstrated that GAITS provides an effective way for team members to monitor and plan the progress of a specific project as well as manage a portfolio of projects. The focus of RADx Tech was speed to market, so unlike POCTRN, the SWAT Teams were responsible for all data input into a team's GAITS site. This allowed teams to focus on their work and not take time to learn and use a new management system, particularly for applicants that were established companies with robust innovation methodologies of their own.

**FIG. 2.**
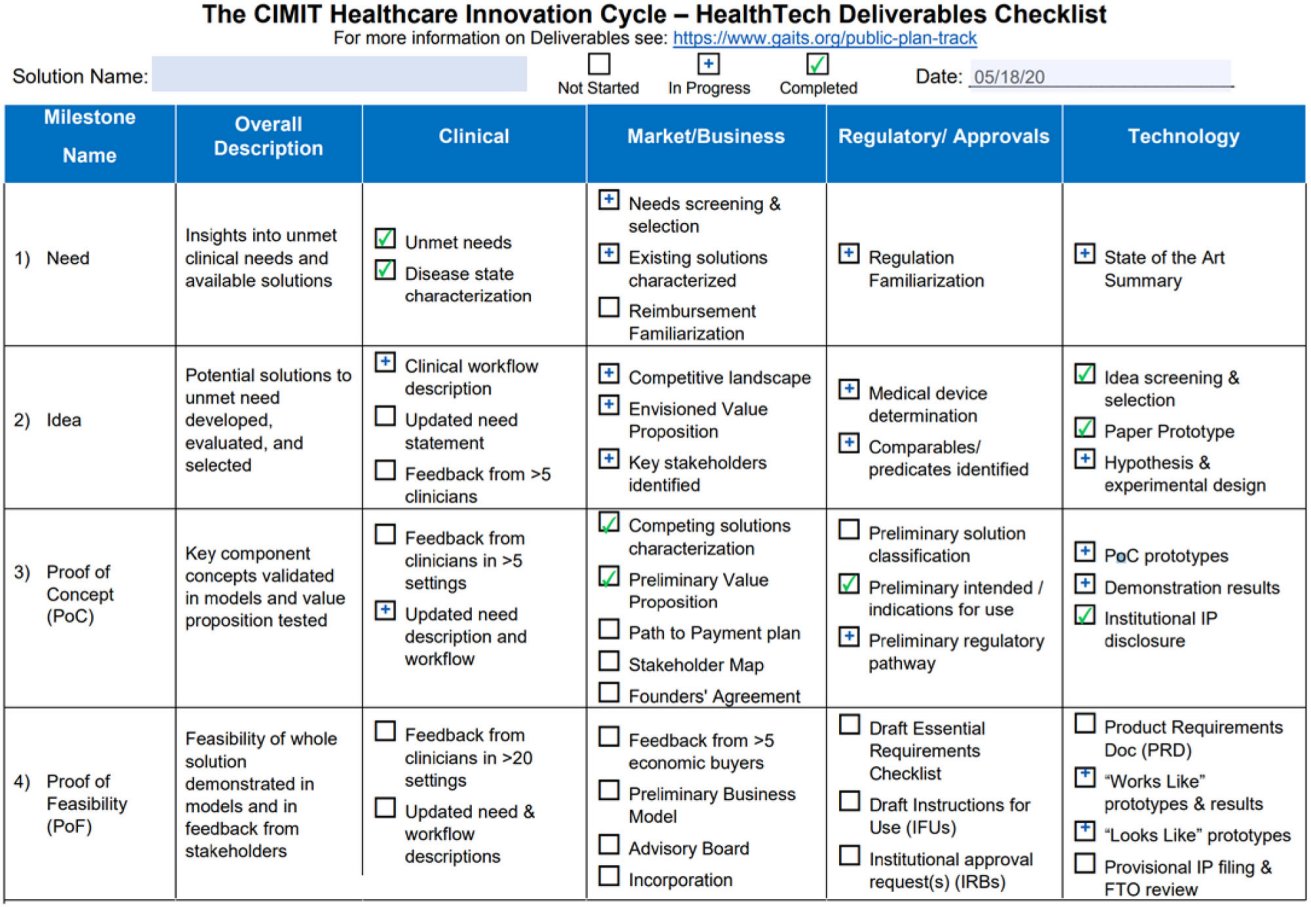
Example Healthcare Innovation Cycle checklist.

*GAITS and the Healthcare Innovation Cycle:* As was done in POCTRN, in the initial application to RADx Tech, teams were asked to complete a checklist of deliverables in the Healthcare Innovation Cycle, which is the foundation for GAITS. This was used as part of the assessment by the Viability Panel to determine if a project was mature enough to move into a Deep Dive. A portion showing the first four maturity levels is shown in Fig. 2.

**FIG. 3.**
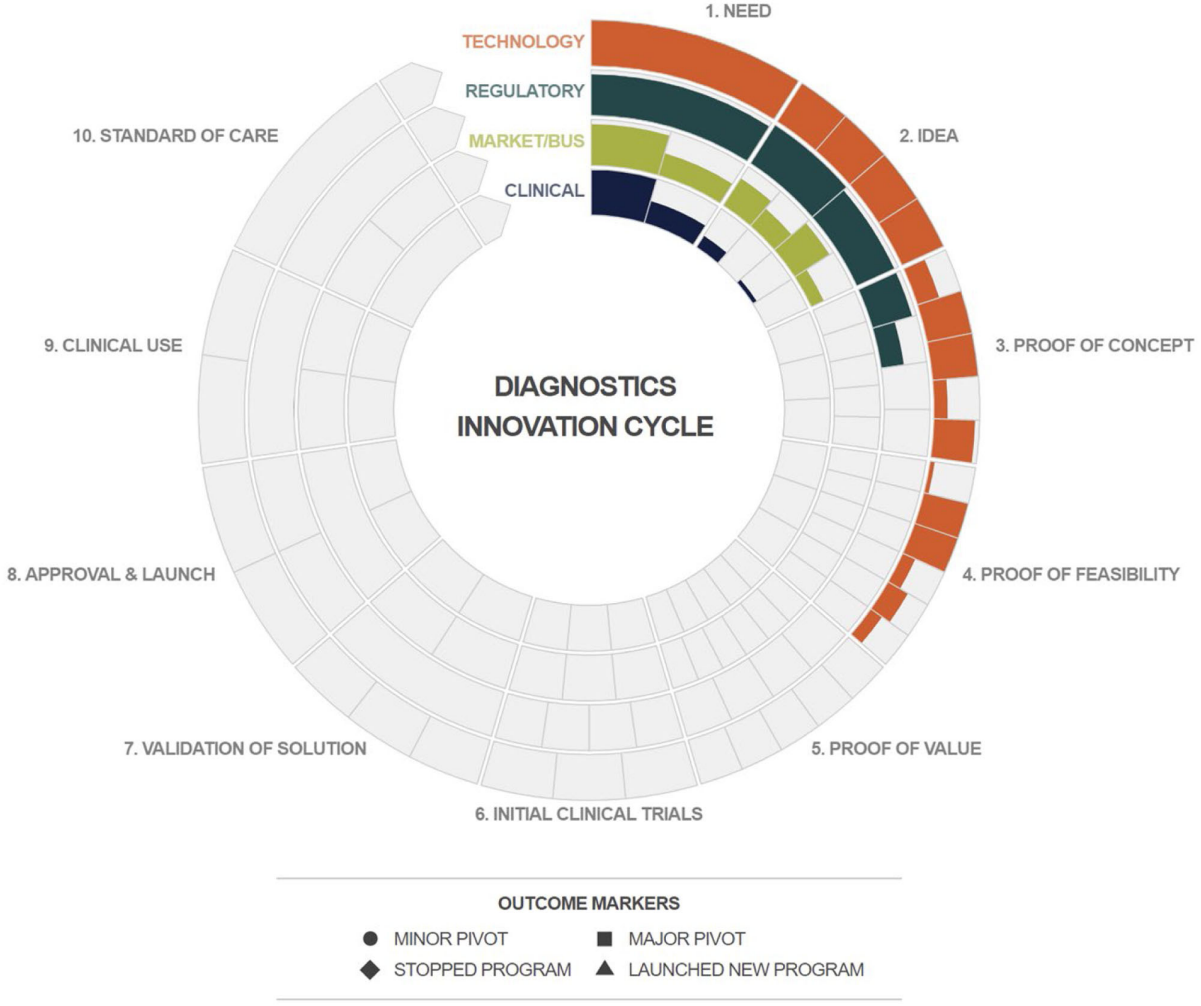
GAITS carousel team self-assessment.

This information was then taken by RADx Tech faculty and entered into a dedicated GAITS site as a team's initial self-assessment. An example is shown in the GAITS Carousel of Fig. 3. The GAITS Carousel provides a snapshot of the work done in a project that quickly conveys the status of a project, with the radial height of each deliverable representing the % complete. The project in Fig. 3 is clearly at risk with the technical work having outpaced the work in the other domains.

One important difference between the checklist and the GAITS sites is that the SWAT teams can define custom deliverables that are specific to a project and/or mark a deliverable as not being applicable. This allows the GAITS site to be very granular in describing the expectations for a specific project.

As projects proceeded into Deep Dive, the SWAT teams update the project status based on its own assessment of a team's progress. With the starting conditions understood, the SWAT team then works with the project team to understand the key risks facing the project in meeting the RADx Tech objectives and developed a two-step plan through to national deployment.

*GAITS Work Packages:* The plans are described through “Work Packages” in GAITS, which are groups of deliverables that make up a series of trackable project go/no-go milestones. These two main Work Packages were then broken down into smaller Work Packages as needed which are used to monitor the progress of a project and its progress against plan.

**FIG. 4.**
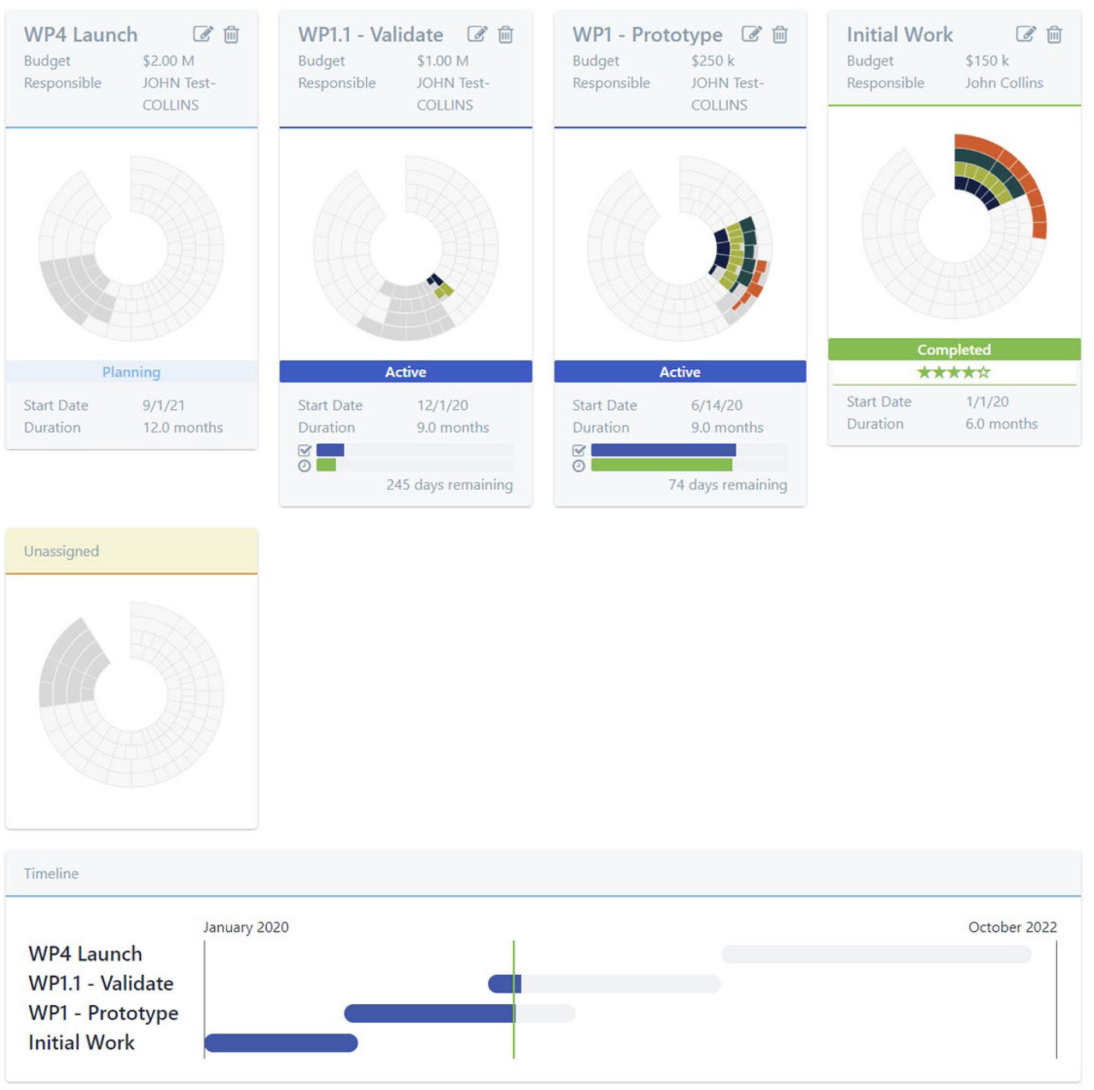
GAITS work package display.

Fig. 4 shows how GAITS displays a hypothetical set of Work Packages (so as not to convey potentially confidential information). The deliverables assigned to a Work Package are highlighted in gray and colored as they are completed. The latest Work Package is in the upper left with the oldest being in the lower right (if there are multiple rows). The Work Package at the bottom of the page shows the unassigned deliverables. The status of work package is shown (planning, submitted, active, or complete). This view for a portfolio manager shows a rating for completed Work Packages, which is not visible to the team. At the bottom is a Gantt view.

This information enables the SWAT Team to quickly convey the status and progress of a project to the RADx Portfolio Managers as well as the Steering Panel and RADx leadership.

**FIG. 5.**
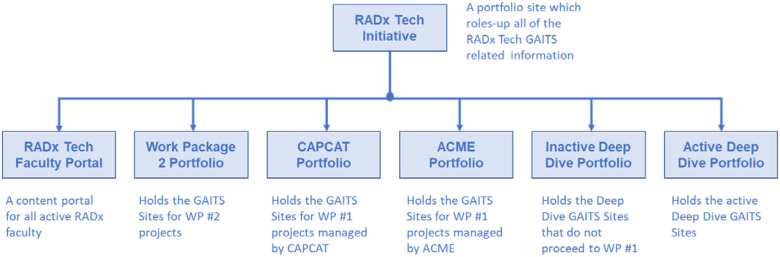
RADx Tech GAITS portfolio site structure.

*Portfolio Sites:* In addition to the individual team GAITS sites are the Portfolio sites. These sites provide access to specific groups of projects by person. Fig. 5 below shows the organization of the RADx Tech Portfolio sites. Note that GAITS sites can be moved between portfolios as management responsibility changes without changing the access by the team members. Like GAITS sites, Portfolio access is at three levels: an admin who can invite other admins, members with read/write privileges, and read-only privileges for observers.

**FIG. 6.**
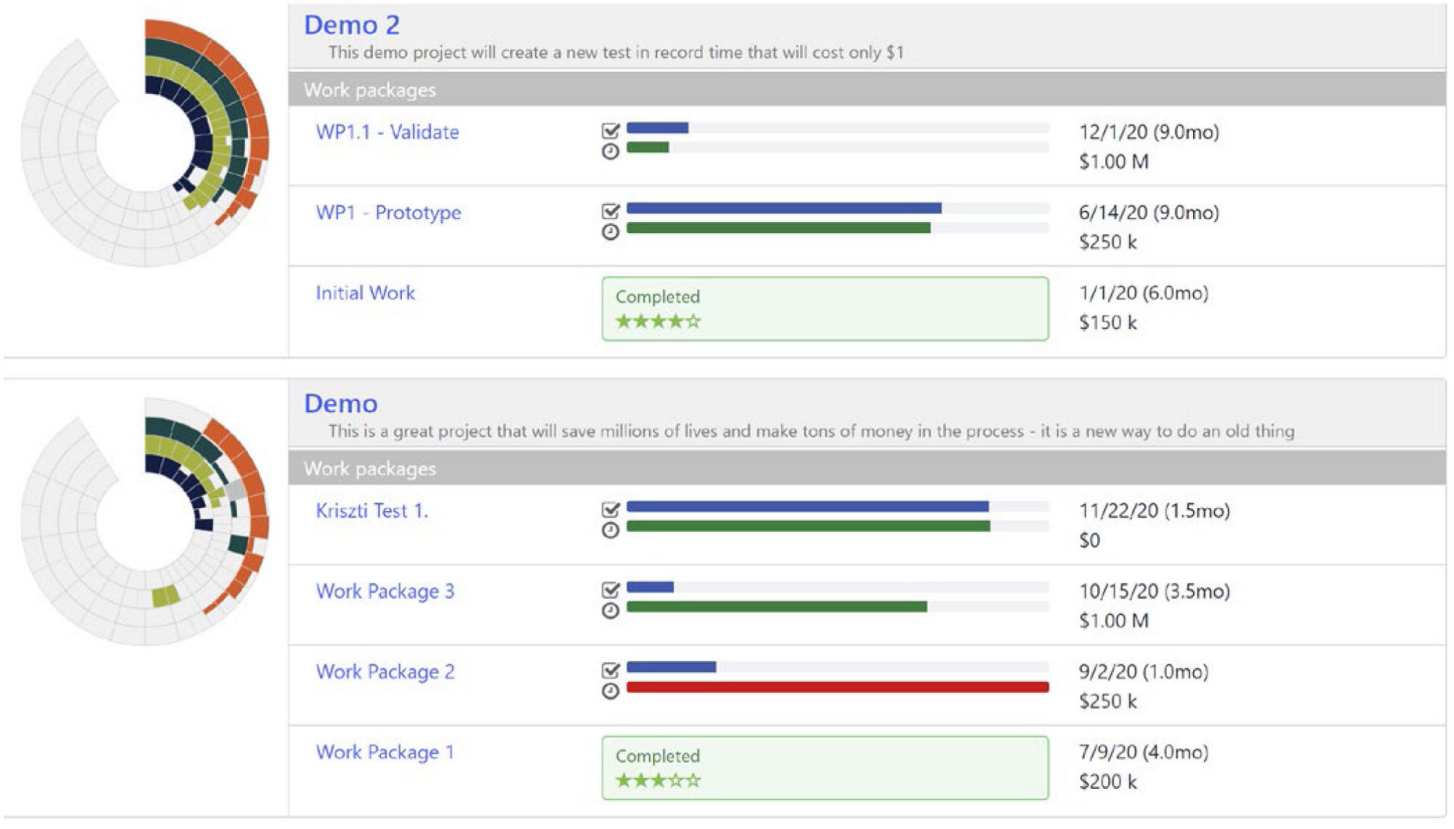
Project portfolio view.

The Portfolio sites are customized but have two important tabs. One tab provides a summary view of each project as shown in Fig. 6 below for a hypothetical set of projects. This allows portfolio managers to quickly scan all the projects to see status and progress against plan for each Work Package.

**FIG. 7.**
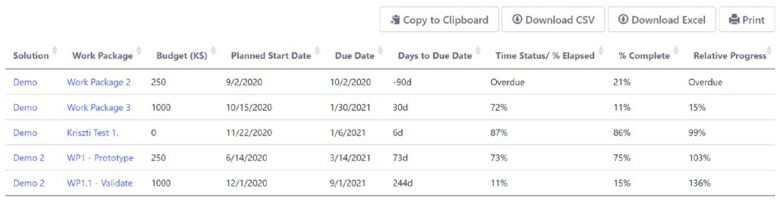
Active work package status tab.

The other tab, shown below in Fig. 7, provides a table that lists just the active Work Packages for each solution (i.e., project or team) in the portfolio. It can be downloaded to Excel and/or sorted to quickly identify problems and/or identify Work Packages that are coming due. This enables managers to quickly assess the status of the portfolio and direct attention where and when needed.

*RADx Tech Faculty Portal:* One portfolio site is very different: the RADx Tech Faculty Portal. It serves as the central hub for RADx Tech programs. The portal was designed to capture and disseminate information securely as well as provide resources to key members of the RADx Tech community. Membership and access are limited to NIH program managers, RADx Tech leadership and central administration (Cores/center leads), SWAT team members, and strategic partners.

The portal functions as a “one-stop-shop” to portfolio and project managers alike. In addition to providing access to the required presentation templates, evaluation criteria, progress reports, and other program-wide materials useful to the experts assigned to specific projects in the Resources section, the site fosters collaboration between RADx SWAT team members across projects and promotes engagement with others within the RADx Tech community with applicable domain expertise to contribute to the program's success by providing a community calendar, directory listing, expert search, and project listing.

The RADx Tech Project Listing is one of the key components of the tech portal. It kept members up to date on the status and progress of each project in the RADx Tech portfolio without listing confidential details on the applicant or the specific type of technology solution in development. For example, the list displays when projects were selected for a stage, track it through the pipeline at each FastTrack decision (Deep Dive, etc.), and lists the SWAT team members. It also records panel review dates and center assignments for advanced projects. The project listing was critical in managing the workload balance across the initiative.

Furthermore, the portal is a tool to coordinate resource allocation toward advanced projects through VentureWell, the clinical cores (see articles by Lam et al. and McManus et al. in this special issue), the large-scale implementation core (see article by Gagliano et al. in this special issue), and administrative centers at Emory (ACME) and UMASS (CAPCaT). Requests to VentureWell and the commercialization sub-core for project management, supply chain/logistics, manufacturing vendors/suppliers, and other critical resources for advanced projects are coordinated through the portal, with links to specific cores meeting request forms, a consultant search, and vendor assignment initiation. The ease of access to RADx cores services, NIH-authorized vendors, domain experts, and consultants enables SWAT teams to quickly close gaps and mitigate device development and deployment risks while working to bring technology solutions to the market in a highly accelerated timeframe.

## V. Challenges and Barriers

There were many challenges and barriers that the RADx Tech program needed to address, particularly given the intense time pressure to produce results while working in the middle of a pandemic. As such, the virtual infrastructure was critical to enable teams with members from around the country that had not worked together before to work in an efficient and structured way. While tools like Zoom, Microsoft Teams, etc. were also critical to facilitate communication, they did not bring the needed structure provided by CoLab and GAITS to ensure that projects were evaluated, run, and managed well.

The challenge presented by the need to start immediately was the lack of time to map out and plan all the needed workflows. The platforms therefore needed to be refined, and in some cases redefined, in real time without shutting down or losing data. Flexibility in being able to configure the platforms to the needs at the time kept the focus on how teams should be working together rather than forcing them to learn and work in a way that the platform was designed to support.

Another challenge represented by the time pressure is that every available minute should be used to advance a project and not spent training in the use of new tools. RADx Tech benefitted greatly from having the CoLab and GAITS usability refined during its previous use in POCTRN so that most tasks were self-evident. The program also invested by having dedicated program experts who were knowledgeable about both the projects and platforms to be available to assist teams as needed.

An increasing challenge facing all virtual infrastructures today is that of security. CoLab and GAITS were built on industry security standards and protocols needed to handle the confidential project data captured. However, as the program progressed and selected projects were successful in transitioning to full development in Work Package 2, the stakes increased by an order of magnitude. It was decided that the level of security needed to increase as well. Therefore, as the program was operating at full speed, the platforms were upgraded to the meet the FIPS 140-2 security standards as well as have in place a System Security Plan (SSP) that followed the NIST SP 800-171 standard. In the case of GAITS, it required changing to the newest and most secure Liferay platform (DXP 7.2) and moving servers to Amazon Web Services.

## VI. Key Lessons Learned

There were and continue to be important lessons learned in using a virtual infrastructure to support accelerated healthcare commercialization programs. Key among them are:

• Use a common innovation framework: This allows diverse teams to start working together quickly and be effective over time. It also streamlines reporting and improves program oversight.
• Capture a longitudinal record: As team members come and go and conditions change, it is often hard to recall what and why some decisions were made. By capturing all the data and key communications in a consistent way, the longitudinal record provides a robust resource.
• Be able to support a desired workflow that may change over time: Too often software platforms are built to support workflows that are commonly used. Instead, platforms should enable users to configure workflows that are appropriate for needs as they evolve.
• Have a small, coordinated core team: RADx Tech relied on a very small core team to drive development and provide support to the diversity of stakeholders. To be effective, they needed to be able to stand-in for each other at a moment's notice while also understanding the work being done by the teams and information needed by RADx Tech faculty and the NIH as well as have mastery of the platforms to spot opportunities for improvement and resolve issues quickly.

## VII. Next Steps

At the time of this writing, the RADx Tech program is ongoing. While the acceptance of new applications is suspended, the supported teams and projects continue to move forward. Fortunately, many projects have already succeeded in developing, producing, and shipping tests because of the support received from RADx Tech in an accelerated time.

One significant benefit of RADx Tech's virtual infrastructure is that all the data generated in the process of selection, management, and monitoring of projects are readily available for reporting and analysis. Work is ongoing to capture output metrics from all the supported projects that can be used to profile results. The data can also be used to conduct analytics and extract lessons learned based on any of the project parameters.

**FIG. 8.**
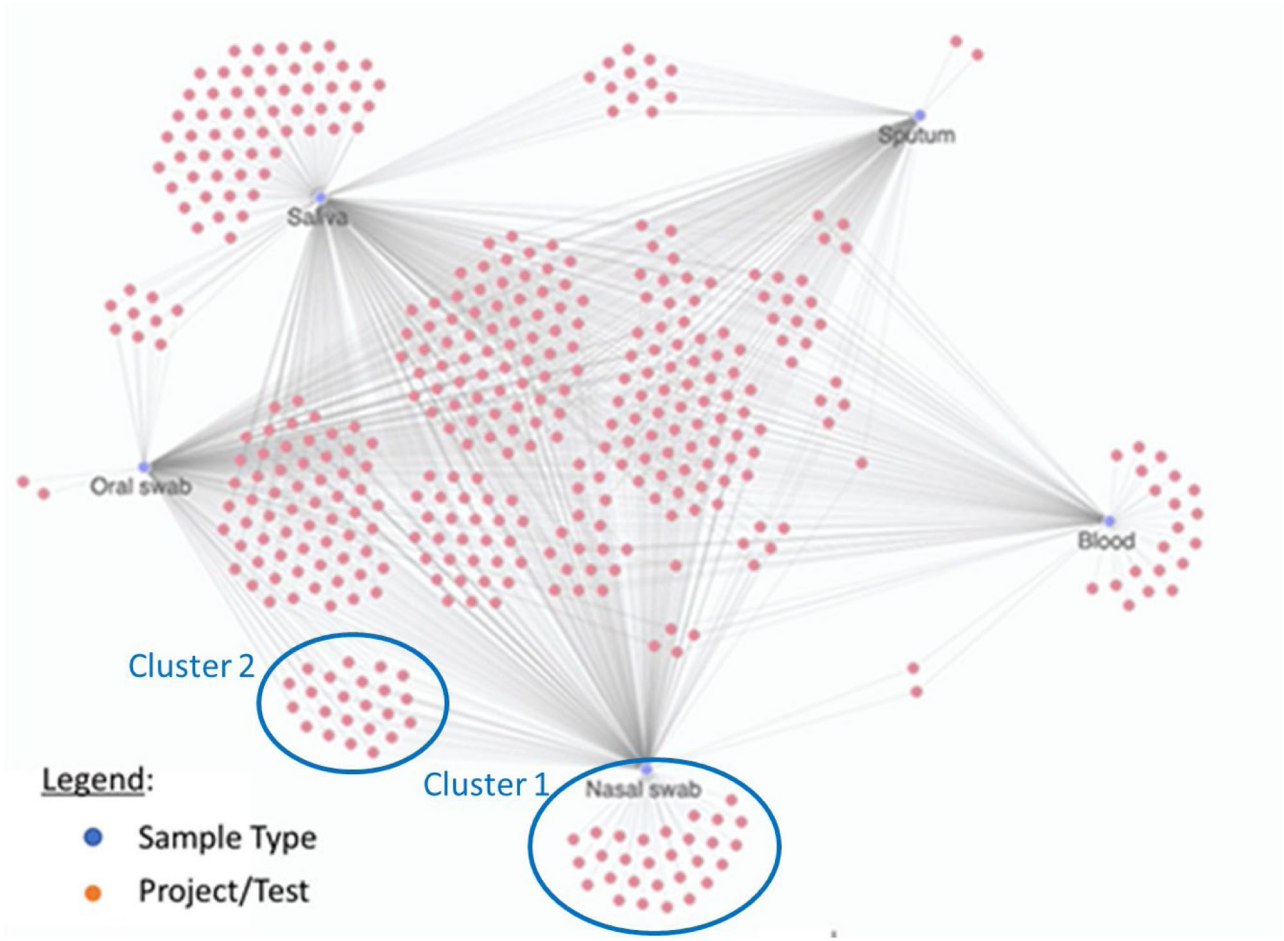
Exaptive sample network diagram.

For example, a series of proof-of-concept experiments were conducted with structured and unstructured CoLab data using the Exaptive visualization and analytics platform^7^. Exaptive uses a patented graph-analytics approach which models complex data as a combinatorial network instead of using traditional row-and-column tables. A simple example is shown in Fig. 8, with a display of the sample types (blue dots) that can used by a test (orange dots). It shows, for example, that the tests in Cluster 1 only work with nasal swabs and that the tests in Cluster 2 work with both oral and nasal swabs. Other Clusters are shown to work with three or more sample types.

Coupling this data with metrics, for example, will enable finding tests that meet certain performance criteria (sensitivity, specificity, etc.) needed for use cases which employ desirable sample types. While simple examples, they begin to show that the number of possible search permutations is enormous. As such, utilizing tools that enable configurable queries to visualize and analyze the data may help in extracting insights.

## VIII. Summary

The CoLab and GAITS web-platforms were critical in being able to manage the RADx Tech's complex workflows involving hundreds of people and teams quickly, efficiently, and effectively. This was a particular challenge for RADx Tech as most of the people had never worked together before. The platforms enabled the selection and acceleration of promising innovative SARS-CoV-2 testing technologies to rapidly develop a nationwide testing capacity.

The platforms also provided the rigor and traceability needed by NIH to ensure that proper controls and approvals were in place to manage work. They provided a framework, proven over CIMIT's 20+ years, that enabled experts to provide support to teams as needed as well as to help teams report progress quickly and in a consistent way to streamline portfolio management.

RADx Tech's web-based infrastructure is scalable and well suited to support other funding programs focused on the commercialization of innovative healthcare solutions. They address the key need for teams to have diversity in expertise while speeding time to patient care. This is likely to increase in importance as innovations today are requiring collaborative teams that have an increasing diversity of technical, clinical, and commercial expertise as well as having members that reside in different organizations and behind separate firewalls. Also, as the rate of technology advancement increases, they help teams accelerate commercialization and meet the objectives of funders with commercialized products and services that result in the improvement of patient care and health.

## Disclosure

The views expressed in this manuscript are those of the authors and do not necessarily represent the views of the National Institute of Biomedical Imaging and Bioengineering; the National Heart, Lung, and Blood Institute; the National Institutes of Health, or the U.S. Department of Health and Human Services.
